# Sound psychometric properties of a short new screening tool for patient safety climate: applying a Rasch model analysis

**DOI:** 10.1186/s12913-023-09768-y

**Published:** 2023-07-10

**Authors:** Niclas Skyttberg, Anders Kottorp, Lisa Smeds Alenius

**Affiliations:** 1 Health Informatics Centre, Department of Learning, Informatics, Management and Ethics, Karolinska Institutet, Solna, Sweden; 2 Faculty of Health and Society, Malmö University, Malmö, Sweden; 3 Medical Management Center, Department of Learning, Informatics, Medical Management and Ethics, Karolinska Institutet, Solna, Sweden

**Keywords:** Patient safety, Questionnaires and surveys, Validation study, Organizational culture, Safety climate

## Abstract

**Background:**

WHO recommends repeated measurement of patient safety climate in health care and to support monitoring an 11 item questionnaire on sustainable safety engagement (HSE) has been developed by the Swedish Association of Local Authorities and Regions. This study aimed to validate the psychometric properties of the HSE.

**Methods:**

Survey responses (*n* = 761) from a specialist care provider organization in Sweden was used to evaluate psychometric properties of the HSE 11-item questionnaire. A Rasch model analysis was applied in a stepwise process to evaluate evidence of validity and precision/reliability in relation to rating scale functioning, internal structure, response processes, and precision in estimates.

**Results:**

Rating scales met the criteria for monotonical advancement and fit. Local independence was demonstrated for all HSE items. The first latent variable explained 52.2% of the variance. The first ten items demonstrated good fit to the Rasch model and were included in the further analysis and calculation of an index measure based on the raw scores. Less than 5% of the respondents demonstrated low person goodness-of-fit. Person separation index > 2. The flooring effect was negligible and the ceiling effect 5.7%. No differential item functioning was shown regarding gender, time of employment, role within organization or employee net promotor scores. The correlation coefficient between the HSE mean value index and the Rasch-generated unidimensional measures of the HSE 10-item scale was *r* = .95 (*p* < .01).

**Conclusions:**

This study shows that an eleven-item questionnaire can be used to measure a common dimension of staff perceptions on patient safety. The responses can be used to calculate an index that enables benchmarking and identification of at least three different levels of patient safety climate. This study explores a single point in time, but further studies may support the use of the instrument to follow development of the patient safety climate over time by repeated measurement.

## Introduction

All around the globe unsafe care is a major contributor to death and disability [1]. In higher income countries one out of ten patients are expected to come to harm in inpatient care [2], up to half of the caused harm is deemed to potentially be preventable [3]. To achieve safer health care delivery the culture of the health care organizations has been increasingly emphasised [4]. Patient safety culture is defined as “a pattern of individual and organisational behaviour, based upon shared beliefs and values that continuously seeks to minimise patient harm, which may result from the process of care delivery” [5]. Culture which are the norms and values of an organization is often studied with methods developed in the ethnographic field of research [6].

Alongside the existing body of literature on patient safety culture, the field of patient safety research also employs the term "patient safety climate" (PSC). Although related, patient safety culture and patient safety climate are distinct concepts in the healthcare domain [7]. Patient safety climate concerns the frontline staff's attitudes towards patient safety in their work environment. It is a narrower aspect of patient safety culture that concentrates on how individuals perceive and understand the patient safety culture within their organization [8].

There is growing evidence to support the correlation between PSC and health care outcomes [9]. Review studies have shown that more than 70% of studies report positive associations between PSC and outcomes in the form of reduced readmissions, length of stay and medication errors [10, 11]. WHO encourages governments around the world to “adopt global approaches for establishment of safety culture across the health system” [1].

In the WHO safety action plan (2021–2030) hospitals are recommended to perform regular surveys of the organization’s PSC [1]. In the Organisation for Economic Co-operation and Development (OECD) working paper on patient safety De Bienassis and Klazinga states that “Without measurement and analysis of the status of PSC in health care settings, it becomes virtually impossible to detect and reinforce beneficial trends that enhance patient safety” [9].

Most studies on PSC have been performed in hospital settings with focus on hospital staff [3]. Fewer studies on PSC have been performed in long term care settings and in primary care [9]. However, the authors of this paper have not identified any studies on PSC in privately owned specialist care provider. Earlier studies have indicated differences in reported patient safety in public and private health care [12] and it is therefore important that instruments are validated in both contexts. Instruments also needs to be validated in both emergency hospitals and planned outpatient care as these contexts may differ in ways of working and experience of PSC.

In 2004 the United States Agency for Healthcare Research and Quality (AHRQ) published a survey on PSC (SOPS®) with an update in 2019. The SOPS® 2.0 reduced the included items from 42 to 32 and the measured dimensions from 12 to 10 [13]. Another survey on PSC is the Safety Attitudes Questionnaire (SAQ) that includes 6 dimensions and 30 items [14]. According to De Bienassis and Klazinga these are the two most widely used surveys for international benchmarking of PSC [9]. However, both surveys are relatively extensive, encompassing ≥ 30 items each.

Survey fatigue is a well described phenomenon describing how respondents tire of answering questionnaires which may cause low response rates and potentially affect validity of the survey [15, 16]. Response rates and the quality of the responses may be affected by the length of the survey, the topic and the complexity of the questions [17].

In addition, surveys with multiple dimensions may come with an inherent risk of “diluting the domain” of PSC [18], consequently lowering the validity and strength of conclusions drawn from the results. The above risks have called for more parsimonious models and shorter surveys in PSC measurements [18].

### The HSE questionnaire

The Swedish Association of Local Authorities and Regions (SALAR) developed an 11-item questionnaire named Hållbart Säkerhets Engagemang (HSE) in 2018 to serve as a quick and efficient tool for PSC screening and benchmarking in clinical practice. It was created to address the need for a short and rapid PSC survey to be used within healthcare organizations in Sweden. The HSE was intended to be used in conjunction with more extensive PSC questionnaires if a more thorough survey was required.

The HSE was piloted in an acute care hospital setting and tested in a confirmatory factor analysis, which showed satisfactory loadings (Danielsson, M, 2022, personal communication, January 12). However, to date there are no published studies on the HSE's validity, reliability, or performance in clinical practice. To address this gap and to further evaluate the instrument, the present study explores the validity and reliability of HSE by applying a Rasch model using data from a privately owned specialist care provider.

### Rasch model analysis

Rasch analysis is a type of psychometric analysis, within the field of item response theory (IRT), that evaluates several aspects of validity evidence, including internal structure, response processes, and fairness in testing [19]. It assesses how well the items in a scale or questionnaire together measure the construct of interest for the target sample, whether the scale scores are related to external criteria or outcomes, and whether the items represent the content domain adequately. Additionally, Rasch analysis [20] can assess the precision/reliability of the scale and provide information on the performance of individual persons as well as items, such as person/item fit, item difficulty, and discrimination. Overall, Rasch analysis can provide valuable information on the psychometric properties of a scale or questionnaire and its suitability for measuring a specific construct [20].

Rasch models are suitable for ordinal scales and have been used for the last decades to develop and validate test and scale construction. The outcomes of Rasch models align well with the concepts suggested for use in scale development and construction [20].

### Aim and research questions

The aim of this study was to explore aspects of validity and precision of a PSC survey, the HSE, designed and developed by SALAR.

Specific research questions, with reference to relevant step in analysis in parenthesis:

1. How are the rating scales used in the HSE functioning? (Step 1)
2. Is there satisfactory evidence of internal scale validity, person response validity and unidimensionality in the HSE*?* (Step 2a-c)
3. How well targeted are the HSE questions to the respondents? (Step 3)
4. Is it possible to separate distinct groups among the respondents, i.e., can the HSE separate respondents into different levels of the PSC? (Step 4)
5. Is there evidence to support that any of the background factors have a systematic impact on the pattern of responses to the HSE questions, i.e., Differential Item Functioning (DIF)? (Step 5)
6. What is the relationship between the HSE mean value index and the Rasch-generated measure?

## Methods

### Sample and setting

Data was sampled within a privately owned specialist care provider organization in Sweden. The organization provides various secondary care services, such as inpatient psychiatric care, outpatient cataract surgery and diagnostic imaging. The organization has multiple locations across Sweden. All employees (excluding HR, finance, and IT departments) in the organization were included in a digital survey using the SALAR HSE questionnaire [21]. A total of 3128 questionnaires were sent out and response rate was 66% giving a total of 2076 responses. It was not possible for responders to complete the questionnaire with missing data and therefore all completed surveys collected data on all 11 questions. The survey results were fed back to the line managers in the organisation in an aggregated anonymized form. The results were used in patient safety dialogs at the units with an aim to develop local improvement plans for patient safety.

Background data was obtained from one of the healthcare organization's providers' Human Resources (HR) systems. Due to a recent merger and acquisition program, it was not possible to link some of the care unit HR systems with the survey, limiting the ability to connect background factors with the survey data. As a result, only respondents who worked in units where a link could be established between the specific HR system and the survey were included in further analysis, resulting in a dataset of 761 total respondents. Table 1 presents the characteristics of these respondents.

**Table 1:** Respondent characteristics

**Gender**	Female		Male	
*n* =	*673*		*88*	
**Employee type**	Manager		Co-worker	
*n* =	*137*		*624*	
**Age groups (yrs)**	< 45	45–55		> 55
*n* =	*233*	*225*		*303*
**Number of years worked at the unit**	< *3*	*3–10*		> *10*
*n* =	*210*	*399*		*152*
**Employee net promotor score eNPS**^**a**^	*Detractors*	*Passives*		*Promoters*
*n* =	*109*	*118*		*76*

^a^*eNPS* Employee net promotor score measurer the likelihood of their employees to recommend the organization as a place to work. Further explained below

In this study, the SALAR HSE questionnaire was administered to gather data from employees within a privately owned specialist care provider organization in Sweden. The questionnaire consisted of eleven questions. This study aimed to investigate whether all eleven items could be included in and utilized for a comprehensive index. By including all items in the common index, it would offer practical advantages from a user standpoint, simplifying the assessment process.

Employee Net Promoter Score (eNPS) is a metric used by organizations to measure the likelihood of their employees to recommend the organization as a place to work [22]. It is calculated by asking employees a single question: "On a scale of 0–10, how likely are you to recommend this organization as a place to work?". Employees who answer with a score of 9 or 10 are considered *promoters*, those who respond with a score of 7 or 8 are considered *passive*, and those who respond with a score of 0–6 are considered *detractors*. The eNPS question was answered yearly by employees across the studied organization.

The HSE questions and the frequencies of responses in the analyzed data set are presented in Table 2.

**Table 2:** Frequency of replies per question and response alternative

Survey Question		I strongly disagree	I disagree	I neither agree nor disagree	I agree	I fully agree
1	My line manager’s boss provides the conditions for providing safe care	5	33	116	294	313
2	In my workplace, we learn from what works well	11	17	97	372	264
3	In my workplace, we always act on the risks we see/identify	4	22	99	359	277
4	In my workplace, improvements are always implemented after negative events	13	40	140	339	229
5	I speak up when I think something is about to go wrong	1	4	15	256	485
6	I am not afraid to talk about my mistakes	2	1	28	282	448
7	I am always well received at my workplace when I need help	5	11	67	278	400
8	At my workplace, we have a well-functioning collaboration with other care units	10	32	197	376	146
9	At my workplace, we adapt the work so that safety is maintained when conditions change	12	32	148	342	227
10	I would feel safe if a close relative was cared for at my workplace	15	25	92	269	360
11	At my workplace, we offer patients to be involved in our patient safety work	34	62	284	252	129

### Development of the HSE questionnaire

The HSE questionnaire was specifically designed in 2018 by the Swedish Association of Local Authorities and Regions (SALAR) to meet the need for a short, rapid, and effective PSC screening tool for safety work and benchmarking in clinical practice within healthcare organizations in Sweden. To ensure the questionnaire's relevance and meaningfulness to clinicians and safety professionals, a multidisciplinary team of professionals with expertise in patient safety and clinical practice was commissioned to develop the instrument (N.B. none of the authors of the study presented here were part of the original SALAR development of the instrument).

The SALAR team drew upon various PSC surveys, including SAQ [14], SOPS [13], and Can-PSCS [18], to select the most appropriate items for the HSE questionnaire. The questionnaire comprises 11 items, all of which are scored on a Likert scale ranging from 1 (“I strongly disagree”) to 5 (“I fully agree”). The items measure agreement with positive safety climate, and there is no reversed scoring of items. The first nine items are intended to be used to calculate a mean value index of PSC for benchmarking purposes over time, while items ten and eleven are designed as outcome measures, according to the guidelines provided by SALAR [21]. Face and content validity of the items was evaluated during the development process and resulted in satisfactory outcomes [SALAR unpublished material].

## Data analysis

### Rasch analysis

The analysis was based on a Rasch rating scale model [20]. The 11 items and the five scale steps were analyzed using the Winsteps® Rasch measurement computer program (Version 5.2.3, Portland, Oregon) [23]. The analysis followed a step-wise consecutive model where the outcomes of each step allow actions to refine the tool for the subsequent steps [24–26].

#### Step 1: Evidence based on rating scale response processes

First, the rating scale functioning of the five-category rating scale was investigated to determine whether (a) the average measures on each item for each category advanced monotonically, and (b) were associated with outfit mean square *(MnSq)* values of less than 2.0 for each of the step calibrations [20]. This step evaluated to what extent all the scale steps in the HSE contributed value to the evaluation of the responses.

#### Step 2a: Evidence based on internal structure (local independence of items)

In the first part of the second step, the Rasch model’s assumption of local independence among the HSE items was explored by monitoring the correlations between the item score residuals [27]. A criterion of a shared variance between item score residuals not larger than 50% (corresponding to a correlation coefficient similar or larger than 0.7 between them) to support local independence among items [28]. This test was used to validate that the questions in the HSE were in fact unique items and that co-variation was acceptable.

#### Step 2b: Evidence based on internal structure (item goodness-of-fit)

The fit of the HSE item responses [20] was also evaluated. An item that did not demonstrate acceptable goodness-of-fit to the model (as evident by more unexpected response pattern across individual scores than expected) was then removed, and the psychometric properties of the remaining items were re-analyzed until all remaining HSE items demonstrated acceptable goodness-of-fit to the Rasch model. A sample-size adjusted criterion for acceptable item goodness-of-fit was set for infit mean square (Infit *MnSq)* values between 0.7 and 1.3 logits [29]. Step 2b evaluated whether all items in the HSE measured the same underlying concept or dimension by re-evaluating the survey until all misfitting items were removed, and ensuring that the remaining items demonstrated acceptable goodness-of-fit to the Rasch model, thereby improving the overall psychometric properties of the survey.

#### Step 2c: Evidence based on internal structure (unidimensionality)

The level of unidimensionality was also evaluated by a principal component analysis (PCA) of the residuals, with the criterion that the first latent dimension should explain at least 50% of total variance, in line with earlier studies [24–26]. The eigenvalue of the secondary dimension (reported as first contrast), with an eigenvalue cut-off of 2.0 or higher was also monitored, to signal a lack of convergence in the data. This approach looked at the variance in the responses that was not explained by the primary dimension and checked whether the remaining variance was due to a secondary dimension or simply random noise.

#### Step 3: Evidence based on response processes (person goodness-of-fit)

The criterion for evaluating person goodness-of-fit was to reject Infit *MnSq* values of 1.4 logits or higher associated with a *z*-value of 2 or higher, accepting that 5% of the sample may by chance fail to demonstrate acceptable goodness-of-fit without threatening evidence of person response validity [30–32]. Step three was introduced to explore how many percent of the individual’s response patterns did not fit the expected Rasch model.

#### Step 4: Evidence based on precision/reliability (separation index)

To determine whether the HSE scale could distinguish respondents demonstrating different levels of PSC, the person-separation reliability index was calculated. The criterion was that the HSE scale should be able to distinguish at least three groups (indicating high, medium, and low levels of PSC), which requires a person separation index of at least 2.0 [33, 34]. The internal consistency/reliability was also assessed with the Rasch-equivalent of Kuder-Richardson Formula 20 or Cronbach Alpha. Evidence of any floor or ceiling effects in the HSE were also monitored, and the targeting of the HSE questions to the respondents was monitored using the Wright map output from the Winsteps program [23].

#### Step 5: Evidence based on response processes (Differential Item functioning)

A Differential Item Functioning (DIF) analysis was conducted to investigate if subgroups in the sample had significantly different responses to items despite equal levels of the underlying trait. DIFs were evaluated across the following subgroups: gender, age, employment time, role within organization, and employee net promotor score (eNPS®) [22]. DIF were analysed using Mantel Chi-Square test for polytomous data with a Bonferroni adjusted p-values of less than 0.01 [35].

Finally, Pearson’s correlation coefficients were used to evaluate the relationships between the HSE mean value index and the Rasch-generated measures of the optimal valid version of the HSE scale. This test was done to explore the reliability of a HSE raw score calculated as a mean value index from the items in the questionnaire.

## Results

The psychometric properties of the HSE questionnaire were evaluated using Rasch analysis. The analysis assessed item performance, fit to the Rasch model, and questionnaire reliability and validity. A summary of the findings is presented in Table 3.

**Table 3:** Statistical approach, Criteria, and Results of the Rasch Analysis of the HSE (*n* = 761)

Psychometric property	Statistical approach and criteria used for analysis	HSE (11 items)	HSE (10 items, item #11 excluded)
**Rating scale functioning**	(a) the average measures on each item for each category advanced monotonically, and (b) were associated with outfit mean square *(MnSq)* values of less than 2.0 for each of the step calibrations	Both criteria met	Both criteria met
**Internal structure**	**Local independence of items** The criteria were set for a correlation coefficient not exceeding .50 to support local independence among HSE items **Principal component analysis of the residuals** The criterion was set for at least 50% of the total variance to be explained by the first latent variable, associated with an eigenvalue of the first contrast (secondary dimension) of less than 2.0	Criterion met Criterion met 52.2% of the variance explained by the first latent variable. Secondary dimension associated with an eigenvalue of 1.75	
	**Item goodness-of-fit statistics** A sample-size adjusted criterion for item goodness-of-fit requiring infit *MnSq* values between 0.7 and 1.3	Item #11 demonstrated misfit (1.42) and was hence excluded	All item fit
**Response processes**	**Person goodness-of-fit statistics** A criterion of < 5% of the sample has unacceptable person goodness-of-fit, as indicated by infit *MnSq* values lower than 1.4 associated with a standardized *z* value < 2.0	-	36 (4.7%)
**Measurement reliability/precision**	**Person-separation reliability** Person-separation index > 2.0 was required to ensure that the scale could differentiate the sample in at least three different levels of PSC **Internal consistency** Person reliability score > 0.7	-	2.19 Ceiling effect 43/761 (5.7%) Floor effect 1/761 (0.1%) 0.83
**Response processes**	**Differential item functioning (DIF)** No item DIF should exceed a criterion of* p* = .01 in relation to: Gender, Age, Time of Employment, Role within Organization, and eNPS	-	Item #7 did demonstrate DIF in relation to Age, where this item was relatively harder to agree with for Age group 45–55, in comparison to both Age group < 45 as well as Age group 55 <

**Fig. 1:**
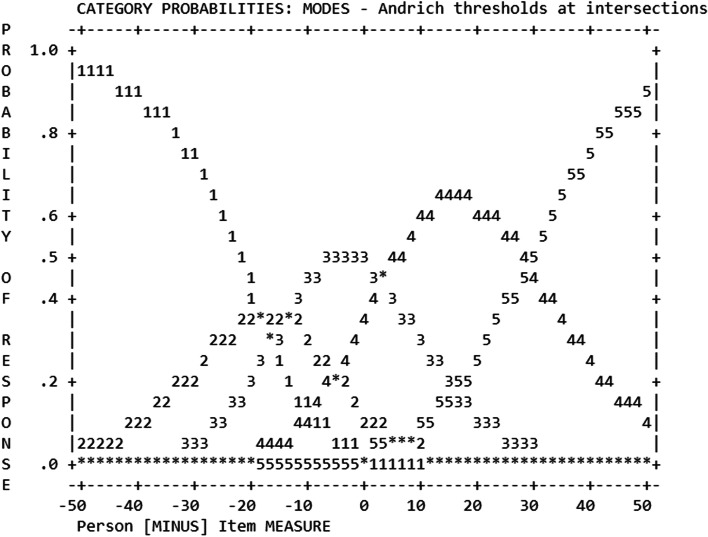
Visual presentation of rating scale functioning with category thresholds for the HSE 11 item version (*n* = 761)

*Step 1: Evidence based on rating scale response processes.* The average measures for the response categories advanced monotonically with an outfit MnSq < 2.0 for all scale steps. However, as the category probabilities curve (Fig. 1) show the category 2 was almost completely covered by category 1 and category 3, the added value of step 2 could therefore be considered limited. As the scale steps met our set criteria, we did not collapse scale steps, but proceeded with the analysis.

*Step 2a: Evidence based on internal structure (local independence of items).* No item residual correlations exceeded our set criterion. The highest residual correlation was found between items #HSE5 and #HSE6, with a coefficient of *r* = 0.29. Hence, we concluded that the 11 HSE items met the Rasch model assertion of local independence.

*Step 2b: Is there satisfactory evidence of internal scale validity*? *(Item goodness-of-fit).* In the first analysis, item #HSE11 demonstrated an infit *MnSq* value of 1,42 and was therefore considered to fit less well than the other items in the HSE scale. When item #HSE11 was removed from the analysis, the remaining 10 HSE items demonstrated infit *MnSq* values between 0.7–1.3. Item #HSE10 demonstrated an infit *MnSq* value of 1.25 when analyzing all 11 items, and 1.29 when item #HSE11 was excluded.

Step 2c: Evidence based on internal structure (unidimensionality). The explained variance was 52.2% which was above the set criterion of 50%. 7.6% of the unexplained variance was attributed to a single contrasting dimension, with an eigenvalue of 1.75. We therefore concluded that there was empirical evidence of unidimensionality in the HSE.

Step 3: Evidence based on response processes (person goodness-of-fit). 36 respondents in our sample (4.7%) were considered giving more variations in responses than expected according to the Rasch model, which was below the set criterion.

Step 4: Evidence based on precision/reliability (separation index). The person separation index of the 10 item HSE scale (excluding item #HSE11) was 2.19, supporting the assumption that the HSE questionnaire could differentiate between at least three different levels of the latent trait (PSC). 43 /651 scored a maximum on all HSE items, giving a ceiling effect of 5,7% of the respondents. One respondent provided minimum scores on all items resulting in a floor effect 0,1%. The person reliability score was 0.83 which met the criterion set to > 0.7.

Step 5: Evidence based on response processes (Differential Item functioning). No items demonstrating significant DIF in relation to Gender, Time of employment, Role within organization, or eNPS. Item #HSE7 did demonstrate DIF in relation to Age, where this item “I am always well received at my workplace when I need help” was relatively harder to agree with for age group 45–55, in comparison to both age group < 45 as well as age group 55 < .

**Fig. 2:**
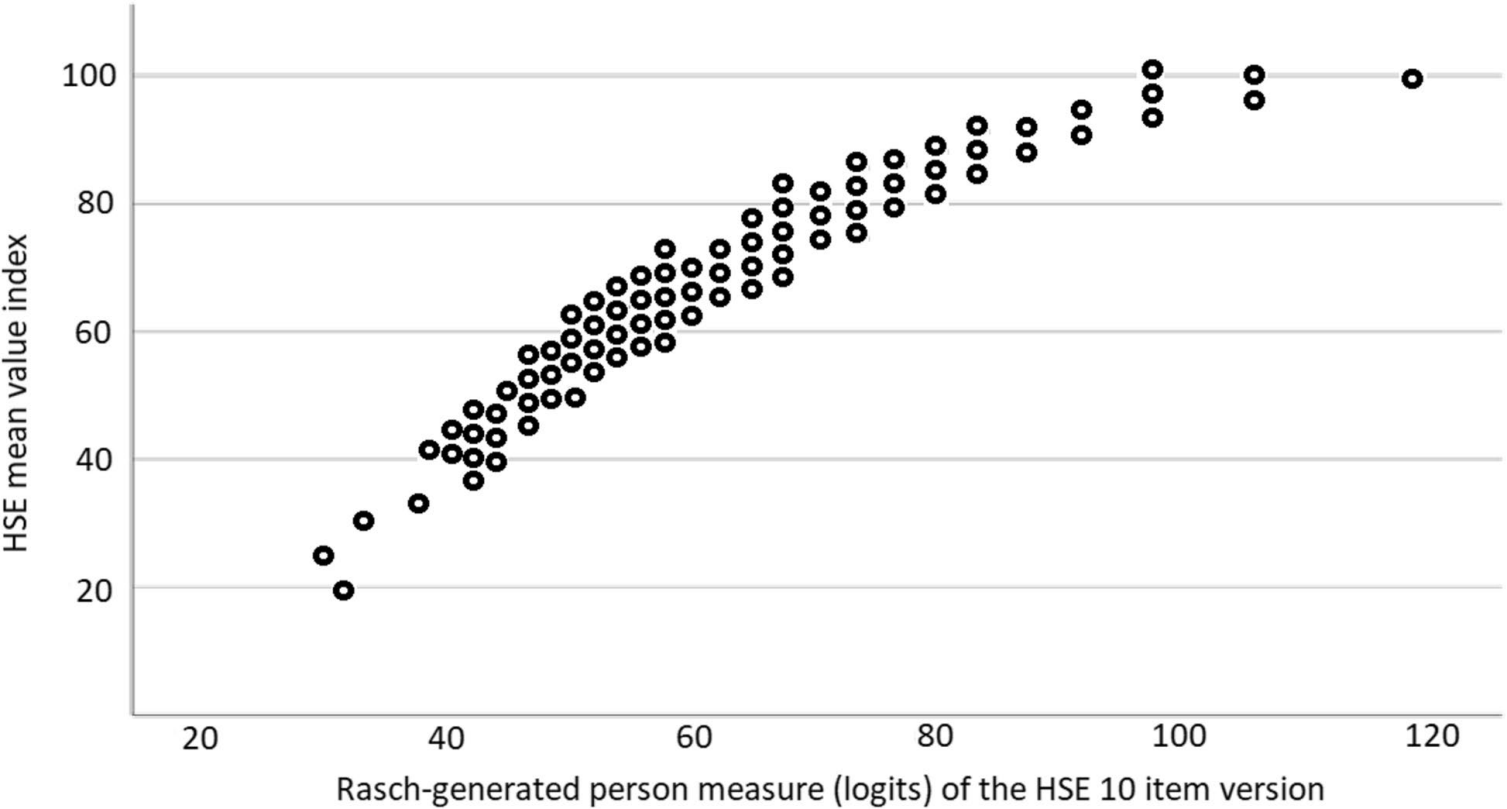
Relationship between HSE mean value indices and the Rasch-generated measures of the HSE 10 item version (*n* = 760; excluding one participant with floor effect), *r* = .95

Finally, the correlation coefficient between the HSE mean value index and the Rasch-generated unidimensional measures of the HSE 10-item scale was *r* = 0.95 (p < 0.01). See Fig. 2.

## Discussion

The present study aimed to evaluate the psychometric properties of the HSE questionnaire in a privately owned specialist care provider. The HSE was originally developed by SALAR in 2018 as a screening tool to assess healthcare staff perceptions of patient safety in acute care settings [21]. However, to our knowledge, no published evaluations of the HSE instrument in settings outside of acute care hospitals have been conducted. Therefore, this study contributes to filling this gap by examining the applicability of the HSE questionnaire in a different healthcare context.

Regarding the rating scale used in the survey, our results suggest the number of scale steps can be reduced from five to four steps without losing any information, as shown in Fig. 1. Specifically, we suggest removing the "I neither agree nor disagree" option (scale step three) from the questionnaire or collapsing it with one of the surrounding rating scale categories. The analysis revealed that this scale step overlaps with the others and does not contribute distinct information.

The results confirmed local independence of all HSE items. However, the study found that the inclusion of item HSE11, which assesses whether patients are offered the opportunity to be involved in patient safety work, was not viable due to high infit statistics [36].

This exclusion may be attributed to a lack of consensus among staff regarding this item, as involving patients in safety work may not be standard practice across organizations [37]. The different interpretations of this item across and between organizations may affect its relationship with the other items in the HSE questionnaire. However, it is important to note the significance of involving patients in safety work, as emphasized by the World Health Organization [1].

With HSE11 excluded the remaining ten items formed a common dimension accounting for more than 50% of the variability in the responses. It is worth mentioning that multiple dimensions are often present in surveys related to PSC. In this context, it is interesting to compare the HSE questionnaire with other widely used international patient safety questionnaires, such as the SAQ and SOPS®, which measure six and ten dimensions, respectively. However, previous studies have shown that further exploration of subdimensions may dilute the intended assessment of the common dimension [13, 18].

The study showed that HSE questions were well targeted to the respondents with less than 5% showing outlier response characteristics. Additionally, there were no flooring effects, and the observed ceiling effect was 5.7%, well below the 15% threshold suggested by Terwe et al. [38]. Further, the HSE questionnaire could separate respondents into different levels of patient safety climate, as indicated by a person-separation index exceeding 2.0 [34].

Interestingly, we found that respondents aged 45–55 encountered greater difficulty in agreeing with statement HSE7, "I am always well received at my workplace when I need help," compared to other age groups. This suggested that this item may pose challenges for this age group. Further investigation is warranted to determine whether this issue stems from the item itself or if it reflects a general difficulty among this age group in seeking help. It is worth considering the real-world findings of communication difficulties between older employees and younger managers, as shown by Kunze et al. [39, 40], as a potential factor influencing these responses.

The analysis revealed a strong linear relationship between the index scores derived from the raw scores of the HSE questionnaire's first ten items and the Rasch-generated unidimensional measure. This indicates that the index provides a reliable measure of PSC, particularly within the range of indices 20 to 70, where the linear relationship is closest. However, caution should be exercised when interpreting index scores outside this range, as they may lead to over- or underestimation of PSC.

These findings contribute to the understanding of the HSE questionnaire's validity and its potential usefulness as a screening and benchmarking tool for assessing PSC in healthcare settings. However, caution should be exercised when interpreting the scores in relation to other patient safety outcomes at a unit [41]. Low scores on the HSE should consider the presence of confounding factors such as poor work environment, job dissatisfaction, and high turnover rates [42]. Further studies are required to explore the identified three different levels of patient safety culture and their potential correlation with other measures of patient safety.

The use of a short questionnaire like the HSE offers several advantages over longer surveys. This study achieved an overall response rate of 66%, which exceeds the average response rate of 45% reported by Zha et al. [43], although it falls short of the 80% response rate often aimed for in federal US studies [44]. The relative brevity of the HSE questionnaire may contribute to higher response rates, as it is easier to complete and lowers the risk of survey fatigue [16, 17]. Additionally, the findings suggest that the HSE can be effectively applied in various healthcare settings beyond acute care hospitals, which is significant considering the predominance of studies on PSC conducted in hospital settings [18].

In conclusion, this study provides valuable insights into the psychometric properties of the HSE questionnaire in a privately owned specialist care provider. The first ten items of the HSE demonstrate good measurement properties, and an index based on these items can reliably assess PSC. However, further research is needed to explore the subdimensions and potential correlations with other measures of patient safety. The HSE questionnaire's brevity and its applicability in diverse healthcare settings make it a useful tool for assessing PSC and identifying areas for improvement.

## Methodological considerations

A strength of this study is that it is set in a privately owned specialist care provider and that it thereby strengthens evidence on validity of the HSE questionnaire. The explored context can be expected to differ from the public emergency hospital environment where the questionnaire was piloted by SALAR [Unpublished data by SALAR], consequently this study adds to the transferability of the instrument. Another strength is that the study included multiple respondent background factors thereby exploring differential item functioning based on these factors. Further, the sample size of 761 respondents is a strength and will provide results within 0.5 logits for item calibrations and person ability measures [45]. A strength of applying the Rasch model in the study is in examining different aspects of validity evidence and that it can predict an individual's performance on a specific criterion or outcome. Another strength of the Rasch model is that it evaluates the internal structure/construct validity by examining whether the items in the questionnaire are measuring a single underlying construct.

The study was performed in a Swedish context with an instrument in Swedish. This may impact generalizability of the results in other contexts. However, the instrument was tested in an acute care hospital context during SALAR’s development process, while this study extends applicability by using a sample from a specialist outpatient care setting. This study does not focus on the content of the questions nor to what extent they are related to the participants’ view of PSC. The study does not rule out that there are dimensions of PSC that are not covered by the instrument. However, the questions were developed by SALAR’s team of patient safety experts and according to their unpublished documentation face and content validity tests showed satisfactory results.

The study's reliance on HR system data to determine eligibility for inclusion in the analysis resulted in a relatively large exclusion of units, which raises concerns about potential selection bias. However, it is worth noting that included as well as excluded units, all provide specialist care, which suggests there may be similarities between the two groups that reduce potential bias. Further research is needed to fully assess the potential impact of the exclusion criteria on the study's findings.

### Future research

This study has validated the HSE questionnaire and shown that it can be used to measure the PSC among individuals. A next step is to understand how the survey can be used to assess the PSC at the unit level since studies indicate that improvement efforts should be directed towards the unit [46]. The Swedish title of the questionnaire suggests that the survey can be used to measure sustainability within the PSC domain [21]. This study does not measure results over time and further studies are required to explore HSE’s ability to measure sustainability of the PSC over time. The sustainability perspective is important because improving PSC cannot be considered a one-off effort; it has been shown to require long term institutional commitment [47].

## Conclusion

This study confirms that the ten first items of the SALAR HSE short questionnaire measures PSC in one common dimension. This study also confirms that the raw score of the first ten questionnaire items can indeed be used to calculate a patient safety index. In the study population it was possible to distinguish at least three different levels of PSC thereby also enabling benchmarking. In the original work by SALAR only nine out of eleven items were considered to fit the index. Our study contrasts that statement giving evidence that ten items fit the Rasch model and can be used to measure a common concept or dimension. The high response rate of 66% indicates that the questionnaire is accepted in a mixed specialist caregiver context. In conclusion, the HSE questionnaire shows sound psychometric results in its current state, and we recommend that the first ten items should be used to calculate a PSC raw score. Further studies can expand knowledge on the ability to assess the sustainability of the PSC over time.

## Data Availability

The datasets used and/or analyzed during the current study available from the corresponding author on reasonable request.
